# Evaluation of community pharmacy technicians’ knowledge, attitudes, and practices about erectile dysfunction and its predictors in Gondar Town: A cross-sectional descriptive study

**DOI:** 10.1016/j.heliyon.2024.e36317

**Published:** 2024-08-17

**Authors:** Assefa Kebad Mengesha, Liknaw Workie Limenh, Wondim Ayenew, Gashaw Sisay Chanie, Abdulwase Mohammed Seid, Melese Legesse Mitku, Mihret Melese, Yibeltal Yismaw Gela, Dereje Esubalew, Alemante Tafese Beyna

**Affiliations:** a Department of Pharmacology, School of Pharmacy, College of Medicine and Health Sciences, University of Gondar, Gondar, Ethiopia; b Department of Pharmaceutics, School of Pharmacy, College of Medicine and Health Sciences, University of Gondar, Gondar, Ethiopia; c Department of Social and Administrative Pharmacy, School of Pharmacy ,College of Medicine and Health Sciences, University of Gondar, Gondar, Ethiopia; d Department of Clinical Pharmacy, School of Pharmacy, College of Medicine and Health Sciences, University of Gondar, Gondar, Ethiopia; e Department of Clinical Pharmacy, School of Pharmacy, College of Medicine and Health Sciences, University of Gondar, Gondar, Ethiopia; f Department of Pharmaceutical Chemistry, School of Pharmacy, College of Medicine and Health Sciences, University of Gondar, Gondar, Ethiopia; g Department of Physiology, College of Medicine and Health Sciences, University of Gondar, Gondar, Ethiopia; h Department of Physiology, College of Medicine and Health Sciences, University of Gondar, Gondar, Ethiopia; i Department of Human Physiology, College of Medicine and Health Sciences, Ambo University, Ambo, Ethiopia

**Keywords:** Attitude, Community pharmacy, Erectile dysfunction, Gondar Ethiopia, Knowledge, Practice

## Abstract

**Background:**

Erection Dysfunction, which primarily affects males older than 40 years, is the inability to regularly produce or retain a penile erection that is strong enough to satisfy sexual activity.

**Objective:**

To evaluate knowledge, attitudes, and practices related to erectile dysfunction and its predictors among community pharmacy technicians in Gondar, Ethiopia.

**Method:**

We conducted a cross-sectional descriptive study on pharmacy technicians in Gondar from June 1, 2022, to August 30, 2022. The study included 165 respondents and utilized a standardized questionnaire with 42 questions to assess their knowledge (18 items), attitudes (5 items), practices (15 items), and demographic characteristics (4 items) regarding erectile dysfunction. We performed both univariate and multivariate analyses on the collected data.

**Results:**

Multivariate logistic regression analysis showed that greater educational attainment was independently correlated with increased knowledge of erectile dysfunction. For postgraduate pharmacy education, the adjusted odds ratio (AOR) was 0.031 (95 % CI: 0.006–0.170, p < 0.001).

Regarding work experience, the adjusted odds ratios (AORs) were as follows: 6.223E-010 (95 % CI: 9.713E-011-3.987E-009, p < 0.001) for 1–5 years, 7.940E-010 (95 % CI: 1.381E-010-4.566E-009, p < 0.001) for 5–10 years, and 6.134E-010 (95 % CI: 1.333E-010-2.824E-009, p < 0.001) for over 10 years.

Additionally, respondents with 5–10 years of work experience had an AOR of 8.129 (95 % CI: 1.476–44.786, p = 0.016), and the gender of the participants had an AOR of 3.399 (95 % CI: 1.239–9.325, p = 0.017), both of which were associated with erectile dysfunction behaviors.

The aggregate ratings indicated that the participants had moderate knowledge, attitudes, and practices regarding erectile dysfunction. The Pearson Correlation Test revealed a significant positive relationship between knowledge of erectile dysfunction and attitude (r = 0.589 and p < 0.001) and practice (r = 0.524 and p < 0.001). Additionally, attitudes and practices showed a significant positive correlation (r = 0.321, p < 0.001).

**Conclusion:**

The study findings showed that pharmacy technicians have a moderate level of understanding, attitudes, and practices regarding erectile dysfunction. The scores for general erectile dysfunction knowledge, attitude, and practice showed a significant positive association (p < 0.001).

## 1 Introduction

An inability to achieve and sustain a strong penile erection for sexual performance is known as erectile dysfunction (ED). Condition must be constant for 3 months to qualify as recurrent [1,2]. It was once thought that ED was primarily psychogenic. Although substantial psychological correlations exist between erectile dysfunction (ED) and clinical comorbidities, which often serve as biological foundations, the latter have gained increasing recognition. The International Society of Impotence Research classifies ED into 3 categories: psychogenic, mixed, and organic. Organic ED includes iatrogenic, neurogenic, vasculogenic, and hormonal types [1,3].

The general consensus is that erections are primarily neurological events involving physiological vascular responses and neurotransmission. Neurovascular regulation also involves biomechanical processes, hormonal inputs, and local biochemical interactions. The field has progressed due to fundamental scientific investigations into the physiology and pathophysiology of the penis, but the identification of clinical illness states linked to ED has also significantly contributed to our understanding of its etiology. When it comes to the vascular relationship, physicians are beginning to see EDs as endothelial dysfunction, which is a vascular disturbance involving the endothelium. Additionally, some risk factors associated with oxidative stress may be involved in the underlying pathophysiology of numerous disease states linked to ED [[4], [5], [6], [7]].

In practice, manifestation, severity, and correlation with specific environmental factors of ED differ between psychogenic and organic categories. Although variations can occur depending on partner and situation, psychogenic erectile dysfunction (ED) typically presents as rapid onset and sudden complete loss of sexual function. In most cases, the patient experiences erection upon waking. Except in cases in which sudden traumatic events cause erectile dysfunction (ED), organic ED typically presents with gradual onset and progressive dysfunction [8,9]. Even during highly stimulating sexual interactions, individuals with biological ED may not consistently achieve erection. Recognizing this distinction can improve ED care plans. When identifying organic etiology, the primary focus shifts to managing comorbid diseases that contribute to the issue [10,11].

The understanding of the prevalence of ED has advanced because of 2 important population-based studies conducted early in the epidemiological examination of condition. 18 % of males aged 50–59 years had ED, according to the National Health and Social Life Survey, which classified different forms of sexual dysfunction in both men and women [12].

About 150 million men face this issue, lowering their quality of life and causing sadness, embarrassment, and feelings of shame, while also leading to significant psychological distress [13,14]. Globally, the prevalence of ED ranges from 2 % in younger men to 86 % in older adults. Regarding the disease's prevalence, there are regional variations [14]. According to previous studies, the prevalence rates of erectile dysfunction (ED) are 80.8 % in Pakistan, 63.6 % in Egypt, and 57.4 % in Nigeria [15]. The Global Online Sexuality Survey reported that 37.7 % of Americans and 45.1 % of Middle Easterners experience ED [16].

Numerous studies have shown a significant association between erectile dysfunction (ED) and factors such as aging, diabetes, obesity, metabolic syndrome, cardiovascular diseases, and benign prostatic hyperplasia [17,18]. Men with diabetes are more than 3 times more likely to develop ED compared with those without diabetes, typically experiencing it 10 to fifteen years earlier. ED in patients with diabetes tends to be more severe, reduces quality of life, and often responds poorly to treatment [19]. Research has also highlighted a strong connection between ED and an increased risk of cardiovascular disease, such as hypertension due to atherosclerosis, and vascular issues, which contribute to higher cardiovascular morbidity and mortality. Additionally, ED is associated with a higher risk of dementia and overall mortality [[20], [21], [22]].

Setting goals for assessment is a crucial first step in managing erectile dysfunction (ED). Clinical decisions should involve both the patient and their partner, considering diverse preferences and expectations for treatment options. Studies have shown that partner interviews influence diagnosis and treatment in 58 % of cases [23,24].

The primary treatment for erectile dysfunction (ED) is phosphodiesterase type 5 inhibitors (PDE5Is), according to the guidelines of the American Urological Association (AUA) [25]. PDE5Is can cause serious adverse events when used inappropriately or poorly. However, they are also very effective and well tolerated [26]. Any PDE5 inhibitor is safe for erectile dysfunction (ED), regardless of whether prescribed on demand or as a daily treatment, and each option has equal safety and effectiveness. Currently, available medications include sildenafil, Tadalafil, and Vardenafil, and newer options include Udenafil, Avanafil, and Mirodenafil. Tadalafil appears to be the most beneficial drug, followed by Vardenafil [27,28]. PDE5 inhibitors increase the risk of melanoma, non-arteritic ischemic optic neuropathy, and prostate cancer recurrence after surgery [27].

Pharmacy technicians are crucial for dispensing medications and providing patient counseling, and their knowledge and practices are essential for the effective management of erectile dysfunction (ED). This study aimed to evaluate the knowledge, attitudes, and practices of community pharmacy technicians regarding erectile dysfunction (ED), identify the factors that influence their understanding and management of ED, and examine how their knowledge and attitudes impact patient care. The following hypotheses were proposed: 1) A significant relationship exists between pharmacy technicians' knowledge of the ED and their attitudes toward managing it; 2) Technicians with more comprehensive knowledge are likely to exhibit better practices in patient counseling and medication management; 3) Predictors such as experience, and educational background are significantly associated with their knowledge, attitudes, and practices. By addressing these objectives and hypotheses, this study seeks to enhance the understanding of pharmacy technicians’ competencies and identify areas for improvement to improve patient outcomes in ED management.

## 2 Methods

**Study Design, Study Period, and Area**: We conducted a cross-sectional descriptive study of pharmacy technicians in Gondar Town, which is situated 734 km northwest of Addis Ababa, the capital city of Ethiopia, in the Amhara Region. The research was conducted from June 1, 2022, to August 30, 2022.

**Source Population**: All pharmacy technicians worked at community pharmacies in Gondar.

**Study Population**: Pharmacy technicians from community pharmacies in Gondar who managed erectile dysfunction medications and agreed to participate in the study.

**Sample Size:** A total of 165 pharmacy technicians were included.

**Fig. 1:**
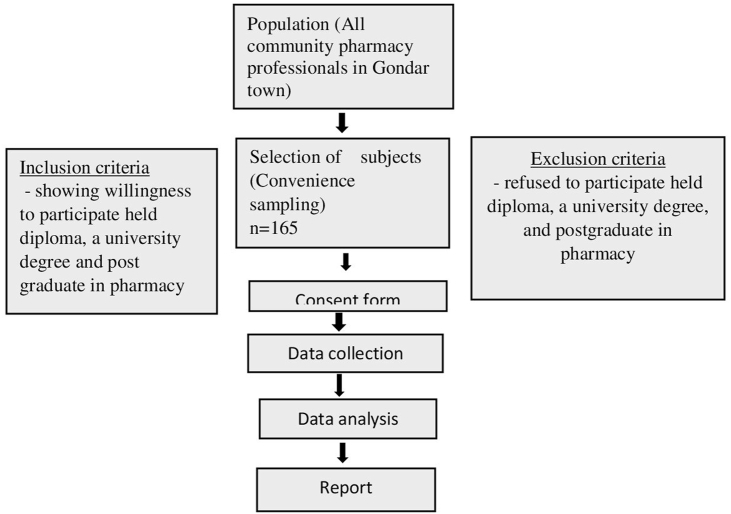
Flowchart of the research methodology.

**Sampling Method**: We used convenient sampling due to its ease, the availability of participants, and the study's time constraints (Fig. 1).

**Investigative Instrument**: To accurately evaluate pharmacy technicians' knowledge, attitudes, and use of erectile dysfunction treatments, we created a questionnaire based on previous studies, which was designed with “Yes,” “I don't know,” and “No” response options. A panel of urologists, pharmacists, and public health professionals reviewed and refined the questionnaire. We conducted face-to-face interviews with community pharmacy technicians to gather information about their knowledge, beliefs, and practices regarding erectile dysfunction and its treatment. We adapted the scoring scheme from earlier research and categorized the overall scores into 3 levels: good, moderate/fair, and poor KAP levels [[29], [30], [31]].

**Fig. 2:**
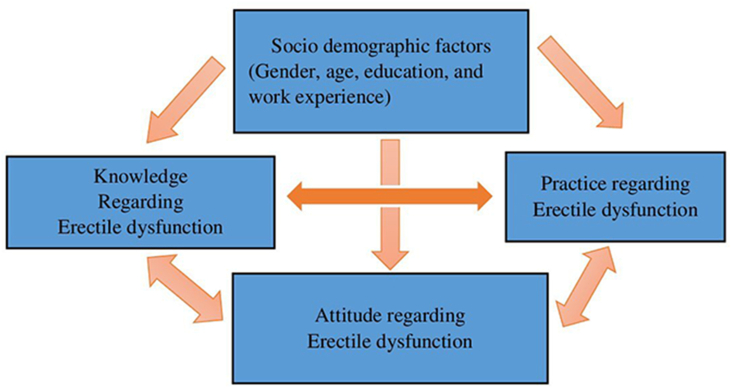
Conceptual framework on the relationship between knowledge, attitude and practice regarding erectile dysfunction and the socio-demographic factors.

**Data Analysis**: We analyzed the collected data using Version 25 of the Statistical Package for Social Sciences (SPSS). For categorical data, we employed descriptive statistics of frequency and percentage, whereas for numerical data, we used the mean and standard deviation (SD). We used a correlation test to evaluate the relationship between 2 numerical variables and compare the overall KAP scores. Both the univariate and multivariate logistic regression analyses examined factors such as age, gender, education level, experience, and knowledge of erectile dysfunction among pharmacy technicians (Fig. 2).

## 3 Results

### 3.1 Socio-demographic characteristics

The study involved 165 participants, with an average age of 31.5515 years (SD = 6.96192). The participants were categorized into 3 age groups: 22–32 years (54.5 %), 33–43 years (33.9 %), and ≥43 years (11.5 %). The sample included 44.2 % women and 55.8 % men. Regarding educational attainment, 46.7 % of pharmacy technicians had a bachelor's degree, 30.9 % had a diploma, and 22.4 % possessed a master's. Work experience varied, with 40.6 % of participants having 1–5 years of experience, 22.4 % less than a year, 20.6 % 5–10 years, and 16.4 % more than 10 years (Table 1).

**Table 1:** Socio-demographic characteristics.

Demographic variables		n (%)
Age	22–32 years	90(54.5 %)
	33–43 years	56(33.9 %)
	≥43 years	19(11.5 %)
Gender	Male	92(55.8 %)
	Female	73(44.2 %)
Level of Education	Diploma	51(30.9 %)
	Bachelor degree	77(46.7%)
	Post-graduate	37(22.4 %)
Level of experience	<1 year	37(22.4 %)
	1–5years	67(40.6 %)
	5–10 years	34(20.6 %)
	>10 years	27(16.4%)

### 3.2 Knowledge, attitudes, and practices (KAP) about erectile dysfunction

The participants had a moderately mean overall knowledge score on erectile dysfunction, which was 37.1333 (SD = 5.32084). According to the survey, pharmacy technicians recognized erectile dysfunction as a complex disease (42.4 %) and observed that it was more common in elderly males (49.7 %). However, the authors also acknowledged that the condition could affect all sexually active men of any age (56.4 %). In young males, psychological problems accounted for 33.9 % of erectile dysfunction cases. The key risk factors for erectile dysfunction in older men are comorbidities, including diabetes, hypertension, and lifestyle choices, particularly smoking (40.6 %, 42.4 %, and 47.9 %, respectively). In contrast, 45.5 % of the respondents recognized that late-onset hypogonadism could cause erectile dysfunction and that testosterone replacement therapy might be necessary. Of the technicians surveyed, 32.7 % stated that oral drugs were the primary treatment option for erectile dysfunction. Of the participants, 41.8 % were aware that PDE5 inhibitors combined with nitrates can cause potentially fatal hypotension.

Mean overall attitude score of 10.8424 (SD = 2.03910) regarding erectile dysfunction, reflecting a moderate level. Most pharmacy technicians (39.4 %) believe that PDE5 inhibitors are suitable for treating erectile dysfunction, a condition frequently recommended by their clients (46.1 %). They were also aware that a prescription is necessary for these drugs (52.7 %) and understood the importance of knowing the quality of medications (27.9 %) due to potential complications (52.7 %).

The mean practice score was 30.0 (SD = 3.6), indicating a moderate practice level. The results revealed that 86 % of technicians sourced ED drugs from retail companies. Between 33.9 % and 49.7 % of technicians conducted quality checks, and 38.2 % combined PDE5 inhibitors with natural remedies like honey. Technicians frequently inquired about co-occurring conditions such as diabetes (46.1 %) and hypertension (47.9 %), and they referred patients to physicians in 41.2 % of cases. Only 46.7 % of patients were prescribed ED drugs without a doctor's approval, and they usually used the same drug and dosage for each patient. Additionally, technicians reviewed the medications taken by patients for these conditions in approximately 60.6 % of cases. Common side effects were headache (53.3 %) and weakness (46.7 %), with sildenafil 100 mg being the most frequently used (60 %) (Table 2).

**Table 2:** Level classification for total scores of knowledge, attitude and practice regarding erectile dysfunction (n = 165).

Percentage of total scores (%)	Total scores of knowledge	Total scores of attitude	Total scores of practice	Level
80–100	39–48	12–14	32–38	Good
61–80	35–39	10–12	30–32	Moderate
0–60	0–34	0–9	0–29	Poor

### 3.3 Univariate analyses

The univariate analysis revealed that male respondents had higher knowledge scores than female respondents (p = 0.085). Higher knowledge scores were also associated with a bachelor's degree (p < 0.001), over 10 years of experience, and being aged ≥43 years (p = 0.008 and p < 0.001, respectively). Additionally, a postgraduate degree (p < 0.010), over 10 years of experience (p = 0.015), and being ≥43 years old (p < 0.0001) were linked to higher attitude scores. Better practice scores were also associated with a bachelor's degree (p = 0.108) and being ≥43 years old (p < 0.042) (Table 3).

**Table 3:** Knowledge, attitudes, and practices scores of the respondents' characteristics and the results of univariate analyses.

Characteristics		Knowledge scores(out of 18) Mean (SD)	P –value	Attitude scores(out of 5) Mean (SD)	p- value	Practice scores(out of 15) Mean (SD)	p-value
Age	22–32 years	1.7333 (0.76143)	<0.001	1.8667(0.62170)	<0.001	1.7556(0.43216)	0.042
	33–43 years	2.2679 (0.79752)		2.2321(0.57179)		1.7321(0.44685)	
	≥43 years	2.4737(0.69669)		2.5263(0.77233)		2.0000(0.000000)	
Gender	Male	2.0978(0.82622)	0.085	1.9891(0.70313)	0.092	1.7826(0.41473)	0.814
	Female	1.8767(0.79835)		2.1644(0.60124)		1.7671(0.42559)	
Education	Diploma	2.1176(0.73884)	<0.001	1.9412(0.67563)	0.010	1.8039(0.40098)	0.108
	Bachelor degree	1.6753(0.81824)		2.0130(0.63853)		1.8182(0.38822)	
	Post-graduate	2.5135(0.60652)		2.3514(0.63317)		1.6486(0.48398)	
Experience	<1 year	1.9700(0.86559)	0.008	1.8378(0.50075)	0.015	1.0000(0.00000)	0.06
	1–5 years	1.8955(0.80027)		2.0149(0.74859)		2.0000(0.00000)	
	5–10 years	1.8529(0.89213)		2.2353(0.49597)		2.0000(0.00000)	
	>10 years	2.4815(0.50918)		2.2963(0.72403)		2.0000(0.00000)	

### 3.4 Factors influencing participants' knowledge, attitudes, and practices regarding erectile dysfunction

Table 4 presents the results of the multivariate logistic regression analysis, examining knowledge, attitudes, and practices as dependent variables. Higher educational attainment was strongly correlated with greater knowledge of erectile dysfunction (AOR = 0.031, 95 % CI: 0.006–0.170, p < 0.001 for postgraduate pharmacy; AOR = 6.223E-010, 95 % CI: 9.713E-011-3.987E-009, p < 0.001; AOR = 7.940E-010, 95 % CI: 1381E-010-4.566E-009, p < 0.001; AOR = 6.134E-010, 95 % CI: 1.333E-010-2.824E-009, p < 0.001 for 1–5 years, 5–10 years, and >10 years of work experience, respectively). The study did not find any significant correlation between the participants' attitudes toward erectile dysfunction and their age, gender, educational attainment, or work experience. However, erectile dysfunction practices were significantly associated with 5–10 years of work experience (AOR = 8.129, 95 % CI: 1.476–44.786, p = 0.016) and female participants (AOR = 3.399, 95 % CI: 1.239–9.325, p = 0.017).

**Table 4:** Factors linked to moderate erectile dysfunction knowledge, attitudes, and practices were analyzed using multivariate logistic regression.

Characteristics		Knowledge score			Attitude score			Practice score		
		AOR	95%Cl	p-value	AOR	Cl 95 %	p-value	AOR	C l95 %	p-value
Age group (ref. 22–32 years^.^)	33–43 years	0.829	0.102–6.750	0.861	2.363	0.376–14.835	0.359	0.184	0.013–2.6840	0.215
	≥ 43	2.095	0.233–18.858	0.510	5.200	0.640–42.277	0.123	0.949	0.057–15.912	0.971
Gender(ref. Male)	Female	1.256	0.454–3.487	0.659	0.490	0.186–1.294	0.150	3.399	1239-9.325	0.017
Education (ref. Diploma)	Bachelor degree	0.229	0.041–1.276	0.093	0.366	0.084–1.597	0.181	0.270	0.042–1.715	0.165
	Post graduate	0.031	0.006–0.170	<0.001	0.415	0.98–1.754	0.232	0.177	0.031–1.011	0.051
Experience (ref. < 1 year)	1–5 years	6.223E-010	9.713E-011-3.987E-009	<0.001	0.596	0.127–2.801	0.513	1.591	0.256–9.875	0.618
	5–10 years	7.940E-010	1381E-010-4.566E-009	<0.001	0.433	0.108–1.730	0.236	8.129	1.476–44.786	0.016
	>10 years	6.134E-010	1.333E-010-2.824E-009	<0.001	4.400	0.401–48.266	0.225	1.500	0.227–9.913	0.674

### 3.5 Relationships among knowledge, attitude, and practice regarding erectile dysfunction

The Pearson Correlation Test revealed a significant positive relationship between knowledge and attitude (r = 0.589, p < 0.001), as well as between knowledge and practice (r = 0.524, p < 0.001) concerning erectile dysfunction. Additionally, the test found a notable positive relationship between attitude and practice (r = 0.321, p < 0.001) (Table 5).

**Table 5:** Association between total scores of knowledge, attitude and practice regarding erectile dysfunction (n = 165).

Variables	r	pP
Knowledge vs Attitude	0.589**	<0.001
Knowledge vs Practice	0.524**	<0.001
Attitude vs Practice	0.321**	<0.001

## 4 Discussion

This cross-sectional study assessed the knowledge, attitudes, and practices of community pharmacy technicians regarding erectile dysfunction (ED) in Gondar, Ethiopia. Additionally, we conducted logistic regression analysis to examine the associations between the pharmacy technicians' age, gender, level of education, and experience with their knowledge, attitudes, and practices concerning erectile dysfunction. Pearson correlation tests were also performed to evaluate the relationships between knowledge and attitude, knowledge and practice, and attitude and practice.

An important aspect of clinical practice is a thorough understanding of PDE5 inhibitors and their adverse effects. Comprehensive knowledge of these medications allows healthcare providers to predict, recognize, and manage adverse reactions effectively, thereby ensuring patient safety and optimizing therapeutic outcomes. Recent studies have highlighted the importance of identifying the safety profiles of PDE5 inhibitors to reduce risks and enhance patient care [32,33].

Despite 42.4 % of our community pharmacy technicians having completed pharmacy and drug education, this level of education has not consistently translated into a comprehensive understanding of ED's causes and treatment. Previous research indicated that although many pharmacy technicians possess basic knowledge regarding ED, significant gaps remain in their understanding of its etiology and current treatment options. This knowledge gap can lead to suboptimal patient counseling and management, underscoring the need for enhanced educational programs. Well-designed training sessions significantly improve both knowledge and attitudes, reinforcing the importance of ongoing professional development to stay current with advancements in ED treatment [26,34].

The current study revealed that 76 % of pharmacy technicians purchase ED drugs from retail companies. Retail companies are a major source of these medications, likely because of their reliability, availability, and ease of access. Such purchasing practices ensure that medications are procured from reputable suppliers while maintaining quality control and regulatory compliance. Additionally, 49.7 % of pharmacy technicians provided prescriptions for ED drugs. Requiring prescriptions aligns with legal and ethical standards and ensures that healthcare providers supervise the appropriate use of medications. This approach helps prevent misuse and allows for effective monitoring of treatment efficacy and adverse effects. A previous descriptive study of 321 questionnaires on the recreational use of PDE5 inhibitors (PDE5i) in healthy young men found that 75.4 % of PDE5i users, mostly those using sildenafil, obtained their medication from friends. Additionally, 17.4 % of patients received PDE5i from pharmacies or drugstores without a prescription, 4.3 % received it from doctors, and 2.9 % received it online [35].

Conversely, 38.2 % of pharmacy technicians reported that they combined PDE5 inhibitors and herbal remedies despite the lack of clinical support for such practices. Previous studies have shown that less than 60 % of non-urologists, especially female doctors, know ED [26]. The use of herbal remedies, such as aphrodisiac herbs, ginseng, yohimbine, and L-arginine, lacks substantial clinical evidence supporting their effectiveness or safety [36]. This highlights the significant need for improved education and training for pharmacy technicians and other healthcare providers. Emphasizing evidence-based practices is crucial for ensuring safe and effective treatment approaches for ED.

A previous study found that over 82 % of physicians referred or consulted patients complaining of ED, indicating a positive attitude toward patient management and typically referring patients to tertiary hospitals for further care [37]. In contrast, only 41.2 % of pharmacy technicians referred ED cases to physicians, whereas 46.7 % provided ED medication only after consulting with a physician. Furthermore, all technicians consistently administered the same type and dosage of PDE5 inhibitors to their clients.

The study reported that 46.1 % of patients with ED had diabetes and 47.9 % had hypertension. Early detection and treatment of these risk factors, particularly hypertension and the dipper pattern, are crucial. Addressing these underlying conditions can significantly reduce the incidence of fatal cardiovascular events among patients with ED [22].

The widespread abuse of pharmaceuticals and the availability of PDE5 inhibitors in community pharmacies across the nation pose serious public health risks. These issues may stem from inadequate drug regulatory frameworks and the need for national medical councils to enforce ongoing drug safety and efficacy evaluations. Community pharmacy technicians often experience underutilization in clinical care, and managing EDs requires a tailored approach rather than a one-size-fits-all solution. Therefore, supporting ongoing medical education and capacity-building initiatives is essential at both the primary and federal levels [38].

Pharmacy technicians with college education benefit from a comprehensive curriculum that covers erectile dysfunction (ED) causes and treatments, providing a solid knowledge base. Their enhanced diagnostic skills allow accurate symptom identification and effective treatment recommendations based on the latest guidelines. Additionally, formal education equips technicians with the necessary skills for better patient counseling and lifestyle advice. Postgraduate education further refines the expertise, providing advanced knowledge, and techniques for managing ED. This background fosters continuous professional development, ensuring that technicians stay updated and deliver high-quality care.

The study found that pharmacy technicians had moderate mean scores for ED knowledge (37.1333), attitudes (10.8424), and practices (30.0). The study found that pharmacy technicians had moderate mean scores for ED knowledge (37.1333), attitudes (10.8424), and practices (30.0). These findings indicate that pharmacy technicians need further education and training in ED management to improve their competency in this area [39].

Given the study's findings of a moderate level of understanding, attitude, and practice among pharmacy technicians regarding erectile dysfunction, coupled with a significant positive association, there is clear room for improvement in the treatment approach.

Enhancing training for pharmacy technicians can deepen their knowledge and improve their practices. Increasing their role in patient education about medication use, side effects, and adherence is crucial. Strengthening collaboration with healthcare providers ensures consistent and accurate patient information. Expanding educational resources, such as brochures and pamphlets, can aid patient understanding. Implementing feedback mechanisms will help identify areas for improvement. Overall, while current practices may be adequate, these adjustments can further enhance patient care and outcomes.

Table 4 provides a comprehensive overview of the multivariate logistic regression analysis, focusing on the relationship between educational attainment, work experience, and knowledge of erectile dysfunction, as well as attitudes and practices among community pharmacy technicians in Gondar Town.

The findings indicated that higher educational attainment is strongly associated with greater knowledge of ED. Specifically, postgraduate pharmacy education showed a significant positive correlation (AOR = 0.031, 95 % CI: 0.006–0.170, p < 0.001). This aligns with recent studies suggesting that advanced education equips healthcare professionals with better knowledge and understanding of specific health conditions, including ED [40,41]. Another study found that postgraduate training significantly enhances healthcare providers' competence in managing and understanding ED [42]. Additionally, work experience also showed a significant correlation, with longer durations of practice (1–5 years, 5–10 years, >10 years) being associated with greater knowledge (AOR = 6.223E-010, 95 % CI: 9.713E-011-3.987E-009, p < 0.001; AOR = 7.940E-010, 95 % CI: 1381E-010-4.566E-009, p < 0.001; AOR = 6.134E-010, 95 % CI: 1.333E-010-2.824E-009, p < 0.001, respectively). Recent literature supports the idea that practical experience significantly contributes to the accumulation of knowledge and skills over time [43,44].

Interestingly, the study did not find any significant correlation between attitudes towards ED and variables such as age, gender, educational attainment, or work experience. This is somewhat consistent with findings from other studies where attitudes toward sensitive health issues like ED are influenced by a complex interplay of cultural, social, and individual factors rather than merely professional background or demographic characteristics [45,46]. For instance, a previous study reported that healthcare professionals' attitudes towards ED were not significantly influenced by their educational background but rather by their personal beliefs and societal norms [45].

When examining practices related to ED, the study found significant associations with 5–10 years of work experience (AOR = 8.129, 95 % CI: 1.476–44.786, p = 0.016) and being female (AOR = 3.399, 95 % CI: 1.239–9.325, p = 0.017). This suggests that those with moderate work experience and female pharmacy technicians are more likely to engage in practices related to ED. This finding can be compared with recent research, which found that mid-career professionals often show more proactive health practices due to a combination of experience and ongoing professional development [47]. Moreover, gender differences in health practices have been documented, with female healthcare providers often demonstrating more comprehensive patient care approaches [48]. Further research is necessary to better understand the impact of gender on knowledge, attitude, and practice.

The Pearson Correlation Test revealed significant positive relationships between knowledge, attitudes, and practices regarding erectile dysfunction (ED). The strong positive correlation between knowledge and attitude (r = 0.589, p < 0.001) indicates that increased knowledge about ED is associated with more positive attitudes towards its management. This relationship is supported by recent studies that emphasize the impact of educational interventions on healthcare providers' attitudes. A previous study demonstrated that educational workshops significantly improved healthcare providers' knowledge and positively influenced their attitudes towards ED treatment [49]. Similarly, another study also found that continuous professional education not only enhanced knowledge but also cultivated a more empathetic and proactive attitude among healthcare professionals towards ED patients [50]. These findings underscore the importance of knowledge acquisition in shaping positive attitudes, which can ultimately enhance patient care.

The significant positive correlation between knowledge and practice (r = 0.524, p < 0.001) suggested that higher levels of knowledge are linked to better practices in managing ED. This aligns with the findings of a previous study, which reported that healthcare providers with greater knowledge of ED were more likely to engage in effective management practices [51]. Furthermore, a previous study highlighted that knowledge gained through targeted training programs led to improved clinical practices and patient outcomes in ED management [50]. These studies highlight the crucial role of knowledge in enhancing practical skills and ensuring high-quality patient care.

The notable positive correlation between attitude and practice (r = 0.321, p < 0.001) indicated that positive attitudes towards ED are associated with better management practices. This relationship is corroborated by recent research emphasizing the influence of attitudes on clinical behavior. For instance, another study found that healthcare professionals with positive attitudes towards ED were more likely to engage in comprehensive and patient-centered care practices [52]. Similarly, a previous study also reported that attitude-focused training programs led to significant improvements in the practical management of ED among healthcare providers [53]. According to KAP theory, higher levels of knowledge can positively influence attitudes, which in turn lead to improved practices and behaviors [54]. Research has supported the idea that KAP-based training can enhance the knowledge, attitudes, and practices related to ED management [55,56].

### 4.1 Study recommendations and their limitations

There are several limitations in our investigation. The straightforward sampling strategy employed in this study, while simple, restricts the applicability of the findings to a wider community. Because of the cross-sectional study design, it was not possible to draw conclusions about the causal relationships between the study's independent variables and outcomes. Random sampling and cohort studies should be conducted to improve results, enhance causal inference, and increase generalizability.

Pharmacy technicians alone will not be sufficient; medical and nursing procedures must also be improved. When legislators, clients, and pharmacists become sufficiently motivated, they will drive these changes. Community pharmacy technicians can elevate ED treatment standards if they work well with men who choose to consult them, in conjunction with other pharmacy staff members and primary and secondary care experts.

## 5 Conclusion

The multivariate logistic regression analysis showed that knowledge of erectile dysfunction (ED) was independently correlated with higher educational levels, particularly among postgraduate pharmacy students, as well as with work experience of 1–5 years, 5–10 years, and over 10 years (p < 0.001). Additionally, there was a strong correlation between job experience of 5–10 years and positive behaviors toward ED, especially among female participants (p = 0.017 and p = 0.016, respectively). However, no significant correlation was found between participants' attitudes toward ED and their sociodemographic characteristics (age, gender, education level, and work experience).

Based on their overall scores, the participants' knowledge, attitudes, and practices regarding ED were moderate. There was a significant positive correlation (p < 0.001) between knowledge, attitude, and practice ratings related to ED. Increased knowledge can positively influence attitudes and practices, thus supporting the KAP (Knowledge, Attitudes, Practices) theory.

The results of this study imply that pharmacy technicians possess the necessary expertise to comprehend the dangers and side effects of pharmaceuticals. Due to their attitudes and knowledge, technicians adhere to standard procedures. Despite this, the findings indicate the need for regulatory measures to promote best practices among pharmacy technicians. Establishing a National Medicine Regulatory Authority could enhance the efficacy, safety, and quality of medications.

This study provides several new insights into the literature by specifically evaluating the role of pharmacy technicians, who often have direct interactions with patients in community settings. This study offers context-specific findings from Gondar, shedding light on regional variations in knowledge, attitudes, and practices regarding ED. By assessing both attitudes and practices, the study revealed gaps between knowledge and practice, highlighting areas for targeted improvement.

Furthermore, the study identified predictors of knowledge, attitudes, and practices that can inform the design of more effective training programs. These findings can enhance pharmacy technicians’ training initiatives, ultimately improving patient care. Increased competency among technicians can improve public health outcomes through safer medication practices and more effective patient counseling.

Overall, this study fills a gap in the literature by highlighting the crucial role of pharmacy technicians in managing EDs and providing actionable insights for their training and practice.

## Ethics approval and consent to participate

The University of Gondar, College of Medicine and Health Science, School of Pharmacy's ethical review board granted ethical clearance (SOP/132/2022).The participants received an explanation of the study's goals and significance. After receiving their informed written consent, the data were gathered, and their names and other personally identifying information were left out, to protect the information's anonymity.

## Consent for publication

Not Applicable.

## Data availability

Data will be made available on request.

## Funding

This research did not receive any specific grant from funding agencies in the public, commercial, or not-for-profit sectors.

## CRediT authorship contribution statement

**Assefa Kebad Mengesha:** Formal analysis, Data curation, Conceptualization. **Liknaw Workie Limenh:** Writing – review & editing, Writing – original draft, Methodology, Investigation, Formal analysis, Data curation, Conceptualization. **Wondim Ayenew:** Wudneh Simegn, Methodology, Data curation, Conceptualization. **Gashaw Sisay Chanie:** Methodology, Investigation. **Abdulwase Mohammed Seid:** Writing – review & editing, Writing – original draft, Validation, Methodology, Investigation, Formal analysis, Data curation, Conceptualization. **Melese Legesse Mitku:** Methodology. **Mihret Melese:** Writing – review & editing, Writing – original draft. **Yibeltal Yismaw Gela:** Methodology, Conceptualization. **Dereje Esubalew:** Data curation, Conceptualization. **Alemante Tafese Beyna:** Writing – original draft, Investigation.

## Declaration of competing interest

The authors declare that they have no known competing financial interests or personal relationships that could have appeared to influence the work reported in this paper.
